# Maternal Obesity Modulates Expression of *Satb2* in Hypothalamic VMN of Female Offspring

**DOI:** 10.3390/life10040048

**Published:** 2020-04-24

**Authors:** Kelly A. Glendining, Lorryn C. Fisher, Christine L. Jasoni

**Affiliations:** Centre for Neuroendocrinology, Department of Anatomy, University of Otago, Dunedin 9054, New Zealand; kelly.glendining@otago.ac.nz (K.A.G.); lorryn.fisher@otago.ac.nz (L.C.F.)

**Keywords:** maternal nutrition, neurodevelopment, developmental programming, ventromedial nucleus of the hypothalamus, glutamatergic

## Abstract

Maternal obesity during pregnancy is associated with a greater risk of poor health outcomes in offspring, including obesity, metabolic disorders, and anxiety, however the incidence of these diseases differs for males and females. Similarly, animal models of maternal obesity have reported sex differences in offspring, for both metabolic outcomes and anxiety-like behaviors. The ventromedial nucleus of the hypothalamus (VMN) is a brain region known to be involved in the regulation of both metabolism and anxiety, and is well documented to be sexually dimorphic. As the VMN is largely composed of glutamatergic neurons, which are important for its functions in modulating metabolism and anxiety, we hypothesized that maternal obesity may alter the number of glutamatergic neurons in the offspring VMN. We used a mouse model of a maternal high-fat diet (mHFD), to examine mRNA expression of the glutamatergic neuronal marker *Satb2* in the mediobasal hypothalamus of control and mHFD offspring at GD17.5. We found sex differences in *Satb2* expression, with mHFD-induced upregulation of *Satb2* mRNA in the mediobasal hypothalamus of female offspring, compared to controls, but not males. Using immunohistochemistry, we found an increase in the number of SATB2-positive cells in female mHFD offspring VMN, compared to controls, which was localized to the rostral region of the nucleus. These data provide evidence that maternal nutrition during gestation alters the developing VMN, possibly increasing its glutamatergic drive of offspring in a sex-specific manner, which may contribute to sexual dimorphism in offspring health outcomes later in life.

## 1. Introduction

During the prenatal period, the developing brain is vulnerable to adverse maternal nutrition. A growing body of research indicates that mHFD and obesity during pregnancy can adversely impact the health of offspring throughout their lifespan, increasing the life-long risk of obesity, metabolic disorders, and cardiovascular disease [1]. In addition to an increased risk of metabolic disorders, offspring also have a greater propensity for poor neurocognitive outcomes, including anxiety and depression [2,3], however, the incidence of these diseases differs for males and females [4]. Interestingly, animal models of maternal obesity commonly report sex differences in the metabolic and behavioral outcomes for offspring [5], such as female specific deficits in glucose homeostasis [6] and a female bias towards anxiety-like behaviors [7,8,9,10,11]. Maternal obesity programming of offspring metabolic disease risk is proposed to involve the development of leptin resistance [12] and deficits in hypothalamic function [13,14,15]. Maternal programming of anxiety in offspring has been attributed to changes in the amygdala [8,9], hippocampus [7,11], and to reward circuitry across multiple brain regions [16]. However, the underlying mechanism of sex differences in developmental programming of disease is unknown.

A brain region with well-documented sexual dimorphism is the ventromedial nucleus (VMN) of the hypothalamus, which has distinct female and male patterns of gene expression, response to hormones, and neuronal circuitry [17,18]. The VMN is known to be involved in the regulation of metabolism [19,20] and anxiety [21,22], and so presents itself as an ideal site to examine sex-specific impacts of maternal obesity on the offspring’s brain. 

The majority of neurons within the VMN are glutamatergic [23,24]. Glutamatergic neurotransmission, both within the VMN and to other interconnected brain regions, is important for the numerous functions it mediates, including glucose homeostasis, metabolism, and anxiety behaviors [21,23]. This led us to the hypothesis that a maternal high fat diet during gestation may disrupt glutamatergic signaling in the VMN of developing offspring in a sex specific manner. Special AT-rich sequence binding protein 2 (*Satb2*) is exclusively expressed by glutamatergic neurons [25], and is highly expressed in the VMN during development [26]. Using a mouse model of mHFD-induced obesity, we performed a qPCR screen to determine whether adverse maternal nutrition alters the expression of *Satb2* mRNA in the developing VMN of GD17.5 offspring. We found mHFD-induced obesity led to significant upregulation of *Satb2* mRNA expression in the hypothalamus of mHFD female offspring in comparison to controls, but this did not occur in mHFD males. Furthermore, we found that *Satb2* transcript upregulation was associated with an increased number of SATB2 immunolabelled cells residing in rostral regions of the VMN in female mHFD offspring. Together, these data provide additional evidence of sex-specific vulnerability of the offspring’s brain to maternal nutrition during development, and identifies the VMN as a novel brain region that may contribute to sex-differences in the developmental programming of both obesity and anxiety. 

## 2. Results

### 2.1. Satb2 mRNA is Upregulated in Mediobasal Hypothalamus of GD17.5 Female Offspring from mFD-Induced Obese Dams 

**Figure 1:**
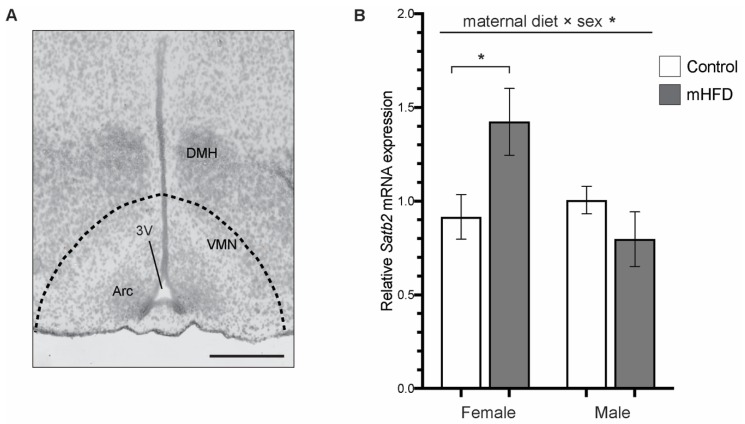
Maternal obesity is associated with upregulation of *Satb2* mRNA in the mediobasal hypothalamus of female offspring at GD17.5. (**A**) Image shows GD17.5 mediobasal hypothalamic region, with the dashed line indicating dissected region containing the arcuate nucleus (Arc) and ventromedial nucleus (VMN) for qPCR analysis. Scale bar is 500 μm. VMN, ventromedial nucleus of the hypothalamus; DMH, dorsomedial hypothalamic nucleus; Arc, arcuate nucleus; 3V, third ventricle. (**B**) Bar graph depicts qPCR analysis of *Satb2* mRNA expression in the mediobasal hypothalamus of control and mHFD offspring at GD17.5. Data are normalized to two validated reference genes and expressed relative to control males as mean fold change ± SEM from *n* = 3–4 independent pregnancies per treatment group, with *p* ≤ 0.05 *.

The quantitative PCR analysis of *Satb2* gene expression in GD17.5 mediobasal hypothalamus (Figure 1A) of control and mHFD offspring, revealed a significant interaction effect between maternal diet and offspring sex [maternal diet × sex *F*_(1, 11)_ = 6.210, *p* = 0.0299] (Figure 1B). 

Post-hoc pairwise analysis indicated a significant >40% upregulation of *Satb2* transcript in the mediobasal hypothalamus of female mHFD offspring, compared to control females (mHFD females: 1.42 ± 0.178, control females: 0.916 ± 0.119, p = 0.0493). In contrast, there was no effect of maternal diet on the expression of *Satb2* in the male offspring’s mediobasal hypothalamus (mHFD males 0.797 ± 0.146, control males 1.01 ± 0.0737; *p* > 0.05) (Figure 1B). 

### 2.2. mHFD Alters the Number of SATB2 Expressing Cells in the Rostral VMN of Female Offspring 

**Figure 2:**
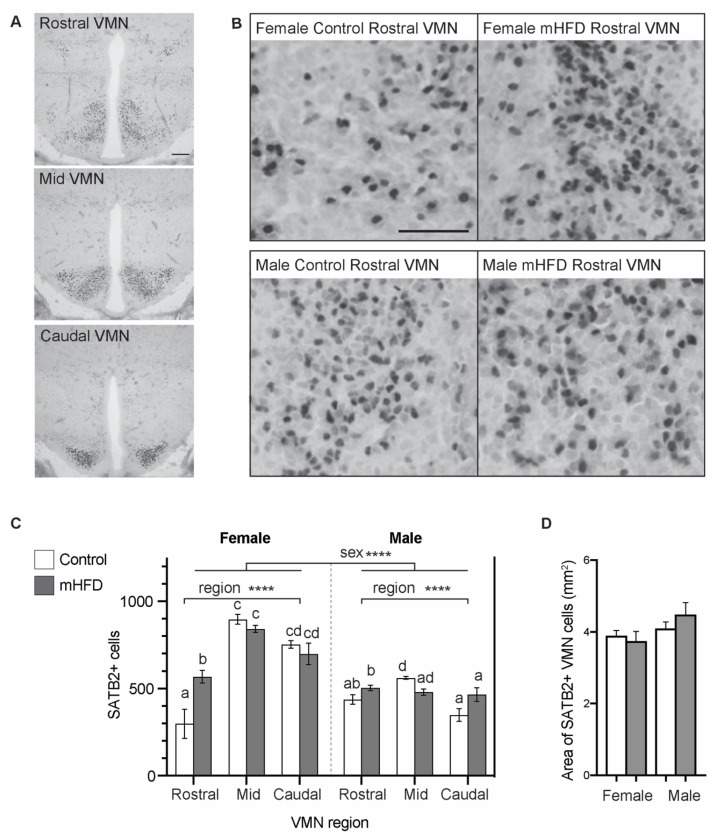
Maternal high fat diet alters the number of SATB2 expressing cells in the rostral VMN of female offspring. (**A**) Image panels show SATB2 labelled cells at representative coronal planes along the rostral to caudal extent of the VMN of the hypothalamus, at GD17.5. Scale bar is 200 μm. (**B**) Image panels showing SATB2 labelled cells in the rostral VMN of female control and mHFD offspring (upper panels), and male control and mHFD offspring (lower panels) at GD175. Scale bar is 50 μm. (**C**) Graph depicts quantification of SATB2 cell numbers at the rostral, mid, and caudal VMN of GD17.5 control offspring (white bars) and mHFD offspring (grey bars). (**D**) Graph depicts quantification of the total VMN area (mm^2^) occupied by SATB2 expressing cells in GD17.5 control (white bars) and mHFD offspring (grey bars). Data are presented as means ± SEMs, with *p* ≤ 0.05 *.

Next, we performed immunohistochemistry to establish whether increased *Satb2* mRNA expression in the mediobasal hypothalamus of GD 17.5 mHFD female offspring, was associated with any changes in the number of resident SATB2-positive cells. Consistent with the *Satb2* mRNA distribution described in neonatal mice [26] and in situ hybridization images from the GD17 mouse brain from Allen’s Developing Mouse Brain Atlas [27], we found SATB2 labelling in the GD17.5 hypothalamus restricted to the VMN (Figure 2). 

We quantified the number of SATB2-positive cells at three coronal planes along the rostral to caudal extent of the VMN (rostral, mid, caudal) (Figure 2A). Analysis of SATB2 cell counts by three-way ANOVA revealed a significant effect of offspring sex on the total number of SATB2 labelled cells within the VMN (*p* < 0.0001; Table 1, Figure 2C), with a greater number of SATB2-positive cells in female VMN than in males. Interestingly, a number of interaction effects between maternal diet, offspring sex, and the VMN region existed, with a significant two-way interaction between the VMN region and maternal diet (*p* = 0.0010; Table 1, Figure 2C), and a complex three-way interaction effect between maternal diet, offspring sex, and the VMN region (*p* = 0.0083; Table 1, Figure 2C). Tukey’s multiple comparisons revealed a significant increase in the number of SATB2-positive cells in the rostral VMN region of mHFD female offspring in comparison to control females (*p* = 0.0023; Table 1, Figure 2B,C), constituting a mean increase in cell number of 90.6% (mHFD female rostral 568 ± 58 cells, control female rostral 298 ± 50 cells). This effect was restricted to the rostral VMN, and maternal diet did not influence SATB2 cell number in the mid or caudal regions of female offspring VMN (mHFD female mid 821 ± 72 cells, control female mid 896 ± 27 cells; *p* > 0.05, mHFD female caudal 684 ± 57 cells, control female caudal 753 ± 45 cells; *p* > 0.05). In mHFD male or control male offspring, there was no significant difference in SATB2-positive cell number in the rostral VMN (*p* > 0.05; Table 1, Figure 2B,C), or in the mid or caudal VMN regions (mHFD male rostral 504 ± 37 cells, control male rostral 438 ± 27 cells; *p* > 0.05, mHFD male mid 479 ± 37 cells, control male mid 561 ± 11 cells; *p* > 0.05, mHFD male caudal 480 ± 35 cells, control male caudal 376 ± 29 cells; *p* > 0.05).

Quantification of total area occupied by SATB2-expressing cells within the VMN indicated that maternal diet was not associated with the expansion or diminution in the distribution of SATB2-positive cells within the VMN [*F*_(1, 8)_ = 0.232, *p* > 0.05] or offspring sex [*F*_(1, 8)_ = 3.659, *p* > 0.05] (Figure 2D).

## 3. Discussion

Here, we report that maternal obesity during gestation leads to sex-specific changes in offspring glutamatergic neuronal cells in the developing VMN of the hypothalamus. The gene expression of the exclusively glutamatergic cell marker *Satb2* [25] is upregulated in the mediobasal hypothalamus of female offspring of obese dams. This was associated with an increased number of SATB2-positive cells in the rostral region of the VMN of female mHFD offspring. In contrast, maternal obesity during gestation does not alter *Satb2* mRNA expression, or the number of SATB2-positive cells in the VMN of male offspring. 

The VMN is a highly interconnected bilateral nucleus within the hypothalamus which sends projections to a multitude of brain regions to regulate complex processes. Glutamatergic neurotransmission within the VMN, via efferent projections to interconnected brain regions, has been shown to be particularly important for the maintenance of glucose homeostasis, and for mediating anxiety [21,22,23,28]. Given the crucial role of VMN glutamatergic signaling in metabolism and anxiety, a greater number of SATB2-expressing cells in the female mHFD offspring VMN may impact these processes. For example, there is a direct link between glutamatergic VMN signaling and anxiety, with direct inhibition of VMN glutamatergic signaling resulting in reduced anxiety in both males and females [21]. Thus, we might predict that an increase in glutamatergic activity in the VMN would be anxiogenic. In animal models of mHFD, anxiety-like behaviors displayed by offspring consistently indicate a female bias [7,8,9,10,11], thus, increased glutamatergic neurons in the VMN of female mHFD offspring could contribute to the female offspring anxiety phenotype. There is also evidence that glutamate signaling in the offspring brain may be particularly susceptible to modulation by adverse maternal nutrition; for example, we have previously reported that a maternal high fat diet leads to increased glutamatergic related gene expression in the amygdala [8], and maternal perinatal high fat has been shown to impact the expression of glutamate signaling-associated genes in the hippocampus [29].

It is unclear whether the increased number of SATB2-positive cells in the rostral VMN of female mHFD offspring indicates aberrant proliferation or differentiation of this specific cell type, or an upregulation of *Satb2* transcript increases SATB2 protein translation within individual cells in the rostral VMN to improve immunohistochemical detection. A co-localization study examining SATB2 immunolabel in concert with additional neuronal markers would also help to clarify whether there is indeed an increased number of post-mitotic, glutamatergic neuronal cells within the female mHFD VMN. However, a caveat of this is that of the known neuronal markers of the VMN during development, there is some degree of variation in their spatial distribution, timing, and duration of expression [26].

It is interesting that the number of SATB2-positive cells in the VMN differed significantly between male and female control offspring, yet no sex-differences were detected in *Satb2* mRNA expression. This raises the possibility that SATB2 protein translation in the offspring VMN may be under the control of sexually dimorphic post-transcriptional regulation. For example, small non-coding mRNAS, or miRNAs are epigenetic regulatory elements which perform fundamental roles in normal development, and can act as post transcriptional modifiers to control gene expression and translation [30]. Evidence suggests that sex differences exist in miRNA expression in the fetal brain of rodents [31], as well as in humans [32,33], and that maternal nutrition can alter miRNA expression in a sex-specific manner [34]. Furthermore, although in the context of osteoblast differentiation, previous studies have identified multiple miRNAs shown to be capable of regulating SATB2 [35,36], thus it is feasible that differential post-transcriptional regulatory activity contributes to SATB2 sex differences in the offspring VMN. Sex specific transcriptional regulation of Satb2 in mHFD offspring may also derive from factors in the maternal circulation that are disrupted by adverse nutrition. For example, in the mHFD model, maternal leptin levels increase in proportion to adiposity [37,38], and excess maternal leptin can cross the placenta and elevate offspring leptin levels [7,39,40,41]. The VMN is a well-established site of leptin and insulin action [20,23], and interestingly, *Satb2* gene expression has been shown to be responsive to leptin [26]. Thus, it is conceivable that elevated leptin could access the offspring VMN, and a higher sensitivity of female VMN to increased leptin via Leptin receptor expression could contribute to *Satb2* expression changes. Indeed, this would align with recent evidence showing that maternal high fat induces the upregulation of the leptin receptor in the developing hypothalamus of female, but not male offspring [42].

Together, these data contribute to the accumulating evidence that adverse maternal nutrition during development can impact the offspring brain in a sex-specific manner. We identify the offspring VMN as a novel brain region that can be affected by maternal diet during development, which as an important regulator of metabolism and anxiety, may contribute to sex-differences in the developmental programming of offspring disease risk. 

## 4. Materials and Methods 

### 4.1. Animals

All experiments and protocols adhered to guidelines proposed by the National Animal Welfare Advisory Committee, NZ, and were approved by the University of Otago Animal Ethics Committee (ET 16/17; 19 February 2018). C57BL/6 mice were housed under 12:12 h light–dark cycles at 21 ± 2 °C, with access to water and food *ad libitum*. For the diet-induced obesity mouse model, 4-week-old females were randomly assigned to the control diet (Research Diets D12450H, 10% kcal fat, 20% kcal protein, 70% kcal carbohydrate), or high-fat diet (HFD) (Research Diets D12451, 45% kcal fat, 20% kcal protein, 35% kcal carbohydrate), with the control and HF diet containing identical sucrose content (17%). Mice were maintained on respective diets for 10 weeks, whereby approximately 60% of HFD mice had bodyweights ≥ 30% than controls (t-test, *p* ≤ 0.05). High fat diet resistant dams were excluded from this study. For mHFD and control offspring, females were timed-mated overnight with 6–8-month-old, chow-fed males, with the following morning deemed gestational day (GD) 0.5. Offspring were collected by caesarean at GD 17.5 between 9:00–10:00, and brain tissue was dissected, flash frozen, and stored at −80 °C. Offspring sex was determined by *Sry* PCR [8].

### 4.2. RNA Extraction and qPCR Analysis

GD17.5 brains were cryo-sectioned at 70 μm, incubated in RNAlater-ICE at −20 °C, and the mediobasal hypothalamus region containing the arcuate nucleus and VMN (Figure 1A) was micro-dissected into Trizol. To ensure there was sufficient RNA for analysis, tissue samples were separated by sex and then pooled so that each (*n*) comprised of four males, or four females, from a single litter. RNA was extracted using the Direct-zol RNA Kit (ZymoResearch, Irvine, CA, USA), treated with DNase I, screened for purity by the NanoDrop ND-1000 (Thermo Fisher Scientific, Waltham, MA, USA) and quantified using a Qubit 2.0 Fluorometer. RNA was reverse transcribed using Quanta qScript XLT cDNA SuperMix (Quantabio, Beverly, MA, USA). A Viia7 Real-Time PCR System was used to perform qPCR under standard thermal cycling conditions. Triplicate reactions (10 μL), contained 2X PowerUp SYBR Green master mix, 1 μL cDNA template and 250 nm forward and reverse primers. Each qPCR run included RNA template controls to confirm the absence of genomic DNA contamination, and no template controls. Primers were designed as previously described [15,43]. Gene expression was normalized to the geometric mean of two reference genes: *TATA box binding protein* (*Tbp*), and *phosphoglycerate kinase 1* (*Pgk1*), which were selected due to stable expression in the GD17.5 mediobasal hypothalamus under different maternal dietary conditions, as determined by Normfinder [44]. Primer sequences used: *Satb2*_F, GTTCGTCTTGGTGCGGAAAG; *Satb2*_R, GGGGCGTCTGTCACATAACT; *Tbp*_F, GAAGAACAATCCAGACTAGCAGCA; *Tbp*_R, CCTTATAGGGAACTTCACATCACAG; *Pgk1*_F, CTCCGCTTTCATGTAGAGGAAG; *Pgk1*_R, GACATCTCCTAGTTTGGACAGTG.

### 4.3. Immunohistochemistry

Immunohistochemistry was performed using standard protocols. Briefly, flash frozen GD17.5 embryo brains were cryo-sectioned at 20 μm, mounted onto gelatinized slides, briefly washed in PBS and fixed in 4% paraformaldehyde in PBS for 10 minutes. Quenching of endogenous peroxidases was performed by incubating sections in 0.3% hydrogen peroxide in methanol for 10 minutes. Sections were then blocked with AffiniPure Fab Fragment Goat anti-Mouse IgG (H + L) at 1:100 in 1% BSA/PBS, 0.3% Triton-X for 2 hours at room temperature. Sections were incubated overnight at 4 °C, with mouse monoclonal anti-SATB2 antibody [SATBA4B10] [Abcam ab51502] at 1:1000. Sections were blocked in 10% goat serum in 1%BSA/PBS, 0.3% triton for 30 minutes at room temperature, followed by biotinylated goat anti-mouse diluted 1:500 in 1%BSA/PBS, 0.3% triton for 2.5 hours at room temperature. For detection of the primary antibody, sections were incubated with avidin-biotin complex (ABC Elite kit, Vector) for 90 minutes at room temperature, and then reacted with 3,3′-diaminobenzidine with nickel enhancer to visualize SATB2 antibody labelling. Finally, sections were dehydrated in a graded series of ethanol, cleared in xylene, and cover slipped with DPX-mounting medium. 

### 4.4. Imaging and Analysis

Digital images of the brain regions of interest for the immunohistochemical analysis were obtained using a Nikon Eclipse Ti2E Inverted microscope, using a 10× objective lens. The distribution of SATB2 cells in GD 17.5 VMN was analyzed at three regions along the rostro-caudal axis (rostral, middle, and caudal levels), with two representative sections imaged at each level with a 10× objective using an Olympus BX51 (Olympus Corporation, Tokyo, Japan). The total VMN area occupied by SATB2 expressing cells was determined by defining the perimeter border of SATB2 immunolabel within the VMN as region of interest at three representative regions along the rostro-caudal axis. Cell counts were performed using ImageJ (version 1.45 s; NIH, Bethesda, MD, USA) as previously described [8]. Briefly, image files were converted to 8-bit, and the background was subtracted using a rolling light function. The perimeter boundary of SATB2-expressing cells within the VMN was defined in binary images using the freehand selection tool, and automated cell counts were performed using the analyze particles function, with circularity set to 50. Initial automated cell counts were performed in tandem with manual counts to confirm accuracy. 

### 4.5. Statistics

For qPCR analysis, the mediobasal hypothalamus samples were obtained from *n* = 3–4 independent pregnancies per treatment group, with each *n* containing pooled tissue from 4 pups within a single litter. Average fold change of gene expression was determined using the 2-ΔΔCT relative quantification method with efficiency correction [45], followed by 2-way ANOVA (maternal diet × offspring sex) and Bonferroni pairwise analysis. SATB2 cell counts were performed on 6 sections from *n* = 3 animals sourced from independent pregnancies per treatment group, and data was analyzed by 3-way ANOVA (maternal diet offspring sex × VMN region), followed by Tukey’s multiple comparison tests. For the VMN area occupied by SATB2 expressing cells, data were analyzed by 2-way ANOVA (maternal diet × offspring sex) and Bonferroni pairwise analysis. All statistical analyses were performed using Prism software (v8.0.2, GraphPad Software, San Diego, CA, USA). Data are expressed as mean ± SEM, and results were considered statistically significant where *p* ≤ 0.05. 

## Figures and Tables

**Table 1:** Three-way ANOVA analysis of SATB2 cell counts in offspring VMN.

ANOVA Table	SS	DF	MS	F (DFn, DFd)	*p* Value	Significance
Region	353,820	2	176,910	F (2, 24) = 39.36	*p* < 0.0001	****
Sex	396,375	1	396,375	F (1, 24) = 88.20	*p* < 0.0001	****
Diet	17,270	1	17,270	F (1, 24) = 3.843	*p* = 0.0617	ns
Region × Sex	277,058	2	138,529	F (2, 24) = 30.82	*p* < 0.0001	****
Region × Diet	84,658	2	42,329	F (2, 24) = 9.418	*p* = 0.0010	***
Sex × Diet	885.1	1	885.1	F (1, 24) = 0.1969	*p* = 0.6612	ns
Region × Sex × Diet	52,894	2	26,447	F (2, 24) = 5.885	*p* = 0.0083	**
Residual	107862	24	4494			

** *p* ≤ 0.01, *** *p* ≤ 0.001, **** *p* ≤ 0.0001, ns *p* > 0.05.

## Abbreviations

3V	Third ventricle
Arc	Arcuate Nucleus
DMH	Dorsomedial hypothalamic nucleus
GD	Gestational Day
mHFD	Maternal high fat diet
qPCR	Quantitative PCR
VMN	Ventromedial nucleus of the hypothalamus
